# Differential Movement Patterns of Juvenile Tengmalms Owls (*Aegolius funereus*) during the Post-Fledging Dependence Period in Two Years with Contrasting Prey Abundance

**DOI:** 10.1371/journal.pone.0067034

**Published:** 2013-07-03

**Authors:** Marek Kouba, Luděk Bartoš, Karel Štastný

**Affiliations:** 1 Department of Ecology, Faculty of Environmental Sciences, Czech University of Life Sciences Prague, Prague, Czech Republic; 2 Department of Animal Science and Ethology, Faculty of Agrobiology, Food and Natural Resources, Czech University of Life Sciences Prague, Prague, Czech Republic; 3 Department of Ethology, Institute of Animal Science, Prague, Czech Republic; University of Lausanne, Switzerland

## Abstract

Fledgling behaviour and movement patterns throughout the post-fledging dependence period (PFDP), especially in relation to changing environmental conditions, have been rarely studied, despite the fact that this period is recognized as of crucial significance in terms of high mortality of juveniles. The PFDP can extend over quite a protracted period, particularly in birds of prey, and a knowledge of the movement patterns of individuals is fundamental for understanding mechanisms underlying survival, habitat use and dispersion. We radiotracked 39 fledglings of the Tengmalm’s owl (*Aegolius funereus*) in two years with different availability of prey: 2010 (n = 29) and 2011 (n = 10) and obtained 1455 daily locations. Fledglings reached independence on average in 45 days after fledging in 2010 (n = 22) and 57 days in 2011 (n = 6). Within years, the most important measures influencing the distance moved from the nest box were age of fledglings and number of surviving siblings present. Individual home range size and duration of PFDP in particular were dependent on maximal number of siblings seen outside the nest box. In the season with low prey availability fledglings were observed at greater distances from the nest box than in the year with higher prey availability (mean distance: 350 m in 2010 and 650 m in 2011) and occupied larger home ranges (mean: 30.3 ha in 2010 and 57.7 ha in 2011). The main factor causing these differences between years was probably the different availability of prey in these two years, affecting breeding success and post-fledging survivorship of the Tengmalm’s owls.

## Introduction

Animal movement is fundamental to many ecological phenomena occurring over a wide range of spatial and temporal scales [1], [2]. At the smallest scale, movements reflect immediate tactical responses to stimuli [3]. At the largest scales, movements are related to dispersal, migration, and colonization [4], [5]. Processes related to foraging, predator avoidance, or mate encounter often occur at intermediate scales [6]. At all scales, movements are constrained by energetic limitations, physical constraints, and behavioural imperatives [7].

In birds the post-fledging dependence period (PFDP) is one of the most sensitive life history stages [8], [9] and movement strategies are one of the primary mechanisms underlying survival during this period [10]. Fledglings of predatory birds are entirely dependent on their parents for food during PFDP and stay within the natal area until the initiation of natal dispersal [11]. This time period is frequently described as most crucial due to incomplete feather growth and inexpert flying skills [12]–[14] and ranges in different species from a few weeks to several months [8].

Detailed studies on movement patterns during PFDP are rare, and those which address owls and raptors have focused mainly on fledgling survival and dispersal (e.g., [12], [15], [16]–[19]). Mortality studies of fledglings have generally considered predation and starvation as the most frequent cause of death [14], [20]–[22]. Other studies have focused on fledgling home range sizes [23] and home range use during PFDP [24]–[26]. Belthoff et al. [23] suggested that juveniles occupied significantly larger home ranges during the latter half of the PFDP as a result of both increased mobility on the part of juveniles and their decreased dependence on the adults [27]. In the Eagle owl (*Bubo bubo*), the mean distance from the nest has been observed to increase significantly with the age of juveniles [28], and average step length, between-sibling distance and post-fledging area gradually increased throughout the PFDP [10]. Despite the fact that fledglings continuously expanded their post-fledging areas, the nest-site still remained the focal point during rearing young and PFDP [10].

During the PFDP parents and offspring may come into conflict over the length of the period of dependency or amount of food delivered [29] and this parent-offspring conflict has been a central topic in evolutionary biology, especially with regard to reproductive effort, parent-offspring communication strategies and PFDP duration [30]–[34]. Variation in prey availability during the PFDP between years should have a strong influence on any parent-offspring conflicts, given that food abundance influences both the condition of the nestlings and the cost of reproduction for the parents [29]. Parent-offspring conflicts should be particularly marked in poor food years because from the chick’s point of view, the length of the PFDP should be maximal in years with low prey availability, as food supply is lower and potentially reduces offspring condition at fledgling [35].

The Tengmalm’s owl (*Aegolius funereus*) is a small, nocturnal, cavity-nesting owl (male body mass c. 100 g), living in coniferous forests in the boreal zone and in alpine forests further south in Eurasia [36]. Hatching occurs at approximately two-day intervals [37]. The young stay in the nest for 28–32 days after hatching [38]–[40], thus fledging at different times, and reach independence 5–7 weeks after fledging [38], [41]–[43]. The great majority of prey brought to the young throughout the late nestling and PFDP in this particular species, is delivered by the male [42]–[44].

The objectives of the current study were to determine movement patterns and home range sizes in Tengmalm’s owl fledglings during PFDP and to estimate the duration of this post-fledging period. We expected that after all individuals fledged from their nest box they would follow the provider, which would be most likely the male [41]–[43], and gradually increase the distance from the nest box. Because the provider would have to move farther to find prey when these are scarce, we would expect the distance between the young and the nest box to be longer and home ranges to be larger when prey abundance is lower. This may be facilitated also by the absence of territoriality in this species [45], because after fledging of the offspring, there is no need for the male to defend the nest-site.

We predicted that (i) juveniles will be found at shorter distances from the nest box and (ii) will occupy smaller home ranges, in years with high prey availability but that both distance from the nest box and home range sizes will increase with the size of successfully fledged sibling flock. Such a result would be expected as a consequence of the fledglings following the male out from the immediate nest-site towards the best hunting area, to decrease prey delivery distance and energy costs of flight. In years of low prey availability a male should forage over a larger area increasing the home range size [46]–[48]; as a result, by moving towards the male the fledglings will also use larger areas and on average be located further away from their nest. Whatever the availability of prey in any season, an effect would be expected in relation to increasing brood size because such broods will need more food items than smaller broods and the provider will also need to forage over a larger area. Such movements may be further exacerbated due to the absence of the female and her help in feeding the young during the PFDP [41]–[43].

We also predicted that (iii) the duration of the PFDP will be shorter in years with high prey availability as a result of parent-offspring conflict [29]. Theory predicts that offspring will be selected to prolong the period of parental care, while parents will stop feeding the young once the cost of parental care surpasses the benefits they obtain in terms of net lifetime reproductive success. This could happen earlier in years with high prey availability since offspring may lose interest in their parents due to better accessibility of prey and due to faster body and feather growth post-hatching and more rapid achievement of adequate hunting skills in rich years [35], [49], [50].

Similarly to results of other raptor studies [50]–[52], we did not expect any effect of individual characteristics (e.g., age at the time of leaving the nest box and body mass just before fledging) on fledgling distances from the nest box, home range sizes throughout the PFDP and PFDP duration. Individual characteristics at the time of leaving the nest box are of less consequence for the period of PFDP as a whole by comparison to the influence of a regular supply of food thereafter; thus these characteristics are likely to be of less significance than that of prey availability through the PFDP.

## Materials and Methods

### Study Area

The study was carried out during two breeding seasons 2010–2011 in an area close to the water reservoir Fláje in the Ore Mountains, Czech Republic (50° 40′ N, 13° 35′ E). This area (75 km^2^, 730–960 m a. s. l.) is now largely forested, with the predominant species being Blue Spruce (*Picea pungens*, occupying approximately 28% of the study area), Norway Spruce (*Picea abies*, 26%), Birch (*Betula* sp., 11%), European Mountain Ash (*Sorbus aucuparia*, 5%), European Beech (*Fagus sylvatica*, 4%) and European Larch (*Larix decidua*, 4%). The area was severely damaged by air pollution in the 1970s, with most coniferous trees above the altitude of 500 m a. s. l. dying out as a result. Out of the forested parts the vegetation is dominated by Wood Reeds (*Calamagrostis villosa*) and solitary European Beech. To compensate for the lack of natural tree cavities, 170 wooden nestboxes lined with wood chips (with the base 25×25 cm, height 40 cm and with an entrance hole 8 cm in diameter) have been installed gradually in the area since 1999.

### Field Procedures

Following the method of Eldegard & Sonerud [41], all nestboxes were visited weekly from early March to find nests and thereafter sufficiently often to check number of eggs and hatchlings and to determine exact hatching date (±1 day). From 25 days after hatching of the first chick, (i.e., shortly before that time when chicks might be expected to leave the nest box), the nest boxes were checked at one or two-day-intervals. All individuals were weighed and the length of wing was measured to estimate the appropriate time for tagging. Owls were trapped and tagged under the Ministry of the Environment of the Czech Republic permit No. 530/758 R/08-Abt/UL and were ringed under the Ringing Centre of the National Museum in Prague permit No. 329.

Fledglings from six nest boxes in 2010 (n = 29) and from five nest boxes in 2011 (n = 10) were equipped with leg-mount transmitter type PIP4 (Biotrack Ltd., UK) about four days before fledging. Transmitters weighed 2.3 g in 2010 and 2.0 g in 2011 (lifespan ±10 weeks) which followed welfare recommendations not to exceed 3% of body mass of tagged individuals (e.g., [53]).

Thereafter, nest boxes were visited at 12–hour-intervals during the night (22∶00–04∶00) and during daylight (10∶00–21∶00) till all siblings fledged and we could determine the exact date of nest box departure. After fledging the young were located once every night by the ‘homing-in’ method [54] till they became independent (i.e., we followed the signal to a particular tree or until we saw the individual). Roosting data were regularly gathered in both years only in first week after fledging (n = 92 locations), thereafter, only occasionally (n = 43 locations). Radio signals were received by using a MVT-9000 receiver (Yupiteru Industries Co. Ltd., Japan) and 3-element Yagi antenna. Fledglings positions (n = 1455 locations in total, 135 of them being gathered during the day) were recorded using the GPS receiver (Garmin GPSmap 60CSx). Fledgling home ranges throughout the PFDP (based on both nocturnal and diurnal locations) were established by the minimum convex polygon (MCP) [55] method generated by Hawth’s Tools (freeware extension for ArcGis, www.spatialecology.com/htools/download.php) and distances between fledglings and their nest boxes were estimated by ArcGis 9.2 software.

PFDP starts with departure of the chicks from the nest but the end of PFDP is less obviously demarcated. We defined the end of PFDP with the first rapid and abrupt movement away from habitual locations [17] which may correspond with cessation of begging for food ([34], this study). These movements were also over a significantly longer distance (>500 m) than movements during PFDP (<300 m). Following the method of Vergara et al. [50] we continued to monitor the locations of all fledglings every night and day for another 14 days in circles up to 5 to 20 km from the last sighting. None of the missing young returned to its natal area. This suggests that the time of independence and time of dispersal is very likely the same in this species.

### Prey Availability

The availability of potential prey items (small mammals: field vole – *Microtus agrestis*, yellow-necked mouse – *Apodemus flavicolis*, bank vole – *Clethrionomys glareolus* and common shrew – *Sorex araneus*) was assessed by snap-trapping in early June in 2010 and 2011. Traps were set up in three hectare squares (with 10 m spacing) located in the study area and were left out for 3 nights, being checked daily after sunrise. The total trapping effort was 1089 trap nights in both seasons. A total of 111 individual small mammals were trapped in 2010 and 6 individuals in 2011, representing 10.2 prey items per 100 trap nights and 0.6 prey items per 100 trap nights, respectively; thus the prey availability was 18.5 times higher in 2010 than in 2011 [56]. Nest box visits showed high numbers of cached prey items throughout the 2010 season (not quantified) and during the onset of breeding in 2011. Later on in 2011 prey availability fell dramatically. In total 22 nestlings starved to death in 2011 (at least five of them even of fledging age).

### Statistical Analyses

We decided that for our purposes in estimating home range, locational fixes do not require serial independence of observations [57]. Instead we followed the approach of De Solla et al. [58] and others (e.g., [59], [60]) of using constant time intervals to maximize the number of observations included in estimations.

Simple comparison of two independent samples was calculated by the Wilcoxon rank sum test (SAS, version 9.3). Associations between the (i) distance from the nest box, (ii) individual fledgling home range size throughout the PFDP, (iii) individual PFDP duration, and other variables (fixed and random effects, see below) were tested using a multivariate General Linear Mixed Model (GLMM, PROC MIXED, SAS, version 9.3) in three separate analyses. To account for the use of repeated measures on the same individuals from the same nest box, all analyses were performed using mixed model analysis with individual fledgling and nesting box as a random factor. Since we expected significant differences between the years, all fixed effects entered the model as nested within the Year (2010 and 2011). We constructed the GLMM entering first expected factor and/or factors and then checking the model with addition of the factors which could also affect the result. The significance of each fixed effect in the mixed GLMM was assessed by the F-test. If not specifically explained, non-significant factors (P>0.05) were dropped from the model and will not be mentioned any further. Where appropriate we tested interaction terms. Associations between the dependent variable and fixed effects were estimated by fitting a random coefficient model using PROC MIXED as described by Tao et al. [61]. We calculated predicted values of the dependent variable and plotted them against the fixed effect with predicted regression lines for each year. Where more than one value was plotted in the same position of the chart, we used a bubble type of the plot.

In all of analyses the following factors were considered as fixed effects: date of hatching, time of fledging and reaching independence; body mass at fledging (g); pooled sibling home range size throughout the PFDP (data from all siblings from particular nest box; MCP, ha); duration of period within the nest box from hatching (days); total number of individuals fledged from particular nest box (1 to 8, “number of fledged siblings”); and mortality rate within the sibling flock (%, the number of dead fledglings as percentage of the total number of fledglings).

In the first analysis (i) distance from the nest box was taken as the dependent variable. Other fixed effects considered in this analysis were time from hatching (27 to 98 days); number of present siblings, which is the actual number of individuals still alive and still dependent seen outside the nest box on any given day (1 to 7); individual fledgling home range sizes (MCP, ha) throughout the PFDP and individual duration of PFDP (days). We also tested the interaction between time from hatching and number of siblings present.

In the second analysis (ii) individual fledgling home range size throughout the PFDP was taken as the dependent variable. Additional fixed effects considered in this case were maximal number of siblings seen outside the nest box, which is the maximal number of live individuals seen outside the nest box after all young had fledged (1 to 7); maximal fledgling distance from the nest box recorded throughout the PFDP (m) and individual duration of PFDP. The home range size of individual fledglings throughout the PFDP showed similar relationships to those for distance of fledglings from the nest box. Since these two variables are inter-related, details of home range size analysis are not presented in the Results section.

In the third analysis (iii) individual PFDP duration was considered as the dependent variable. Other fixed effects considered there were maximal number of siblings seen outside the nest box; maximal fledgling distance from the nest box recorded throughout the PFDP and individual fledgling home range sizes throughout the PFDP. We also tested the interaction between mortality rate and maximal number of siblings.

## Results

### PFDP Duration and Home Range Sizes

Nestlings equipped with transmitters fledged between 23 May and 12 June (median 2 June) in 2010 and between 14 May and 10 June (median also 2 June) in 2011 (Table 1). Fledglings reached independence in 45±4.8 days after fledging in 2010 (n = 22) and 57±3.1 days in 2011 (n = 6) with the range 34–51 days and 53–61 days respectively (Wilcoxon rank sum test: Z = 3.68, p<0.0001). They became independent between 5 and 30 July (median 15 July) in 2010 and between 11 July and 3 August (median 28 July) in 2011.

**Table 1:** Results of breeding data of the studied population.

Year	2010	2011
First eggs in all nests were laid between dates	25 March & 12 June	13 March & 14 May
First egg laid (median)	30 March	6 April
Number of nests	12	24
Number of successful nests (where at least one nestling successfully fledged)	8	8
Number of eggs (mean ± SD)	6.8±1.1	3.7±1.5
Number of hatchlings (mean ± SD)	6.1±1.5	1.9±2.0
Number of fledglings per initiated nest (mean ± SD)	3.9±3.0	0.7±1.1
Number of fledglings per successful nest (mean ± SD)	5.9±1.5	2.0±1.0

Average home range size of individual fledglings during the PFDP was 30.3±16.0 ha in 2010 (range 5.3–61.1 ha) and 57.7±26.7 ha in 2011 (11.9–97.1 ha) – (Wilcoxon rank sum test: Z = 2.21, p<0.01). These MCP ranges were based on 47±8 locations (pooled nocturnal and diurnal locations) on average (range 36–65).

In 2010 fledglings were often silent and continuous use of the radio-receiver to locate them was necessary on nearly every occasion. In contrast, fledglings were calling constantly almost every night during 2011, begging for food (Kouba, unpublished data). Radioequipment was thus in many cases used just for determination of general direction of the fledglings, thereafter locating them by sound in order to get close to them, finally identifying individual fledglings again by radioequipment. It is apparent that each fledgling was receiving less prey relative to its need in 2011 than in 2010.

### General Movement Patterns during PFDP

**Figure 1:**
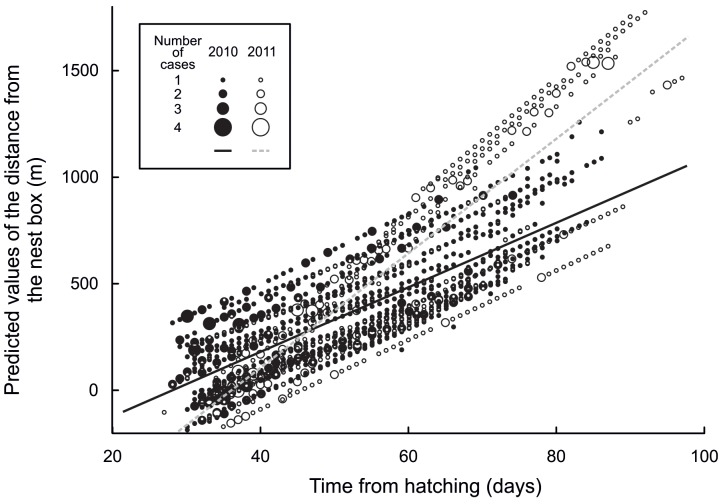
Predicted values of the distance from the nest box for 2010 (filled circles) and 2011 (open circles) plotted against the owlets age from hatching.

**Figure 2:**
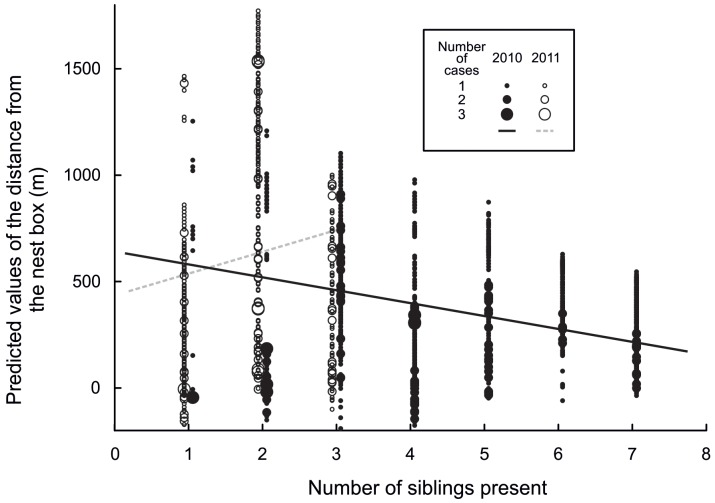
Predicted values of the distance from the nest box for 2010 (filled circles) and 2011 (open circles) plotted against the number of siblings present.

**Figure 3:**
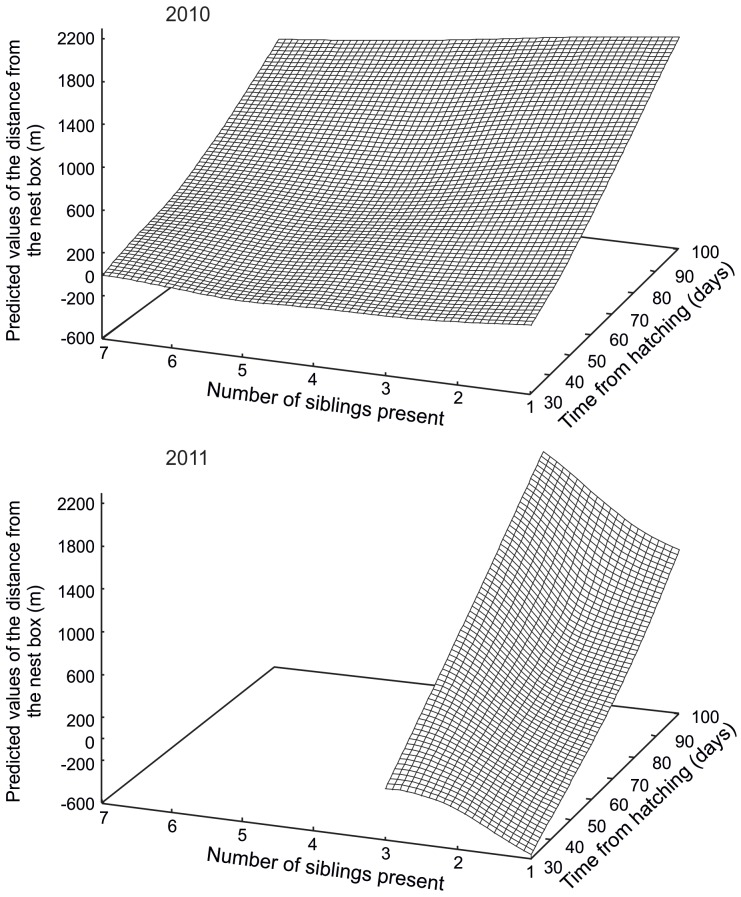
Predicted values of the distance from the nest box for 2010 (top) and 2011 (bottom) plotted against the number of siblings present and the time from hatching.

**Figure 4:**
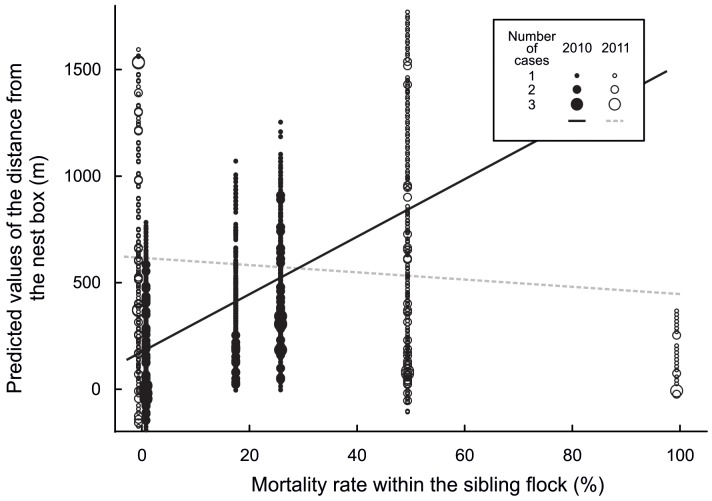
Predicted values of the distance from the nest box for 2010 (filled circles) and 2011 (open circles) plotted against the mortality rate (%, the number of dead fledglings as percentage of the total number of fledglings) within the sibling flock.

The results of the GLMM for the distance from the nest box (Table 2) revealed that the distance from the nest box was dependent on age nested within the year (Fig. 1), number of siblings present, nested within the year (Fig. 2), interaction between age and number of siblings present, nested within the year (Fig. 3) and the mortality rate within the sibling flock nested within the year (Fig. 4). Although the effect of mortality did not reach formal level of significance, we left it in the model which was then offered a better fit as regards to Akaike’s, Schwarz’s and a finite-sample corrected Akaike Information Criterion.

**Table 2:** The results of the GLMM for the Distance from the nest box.

Fixed effect	Num DF	Den DF	F value	P<
Time from hatching nested within the year	2	1158	91.46	0.0001
Number of present siblings nested within the year	2	1062	94.98	0.0001
Interaction between time from hatching and number of present siblings nested within the year	2	1235	92.26	0.0001
Mortality rate within the sibling flock nested within the year	2	9.75	3.54	0.07

Once all individuals from a nest box had fledged, the distance of fledglings from the nest box increased with increasing age (Fig. 1). Distance from the nest box was negatively related to the total number of siblings present in 2010 but positively dependent on sibling number in 2011 (Fig. 2). The interaction between age and number of siblings present was less pronounced in 2010 (Fig. 3 top) than in 2011 (Fig. 3 bottom). Mortality rate within the sibling flock was associated with the distance from the nest box. As distance from the nest box increased, mortality increased in 2010 but remained stable in 2011 (Fig. 4).

**Figure 5:**
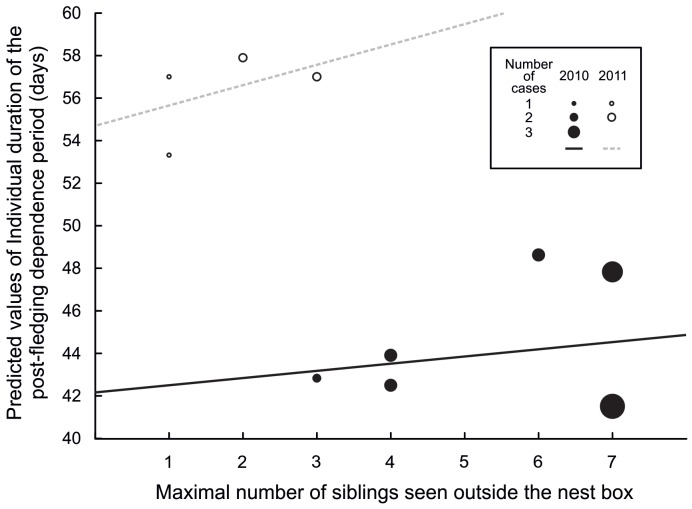
Predicted values of the individual duration of the post-fledging dependence period for 2010 (filled circles) and 2011 (open circles) plotted against the maximal number of siblings seen outside the nest box.

**Figure 6:**
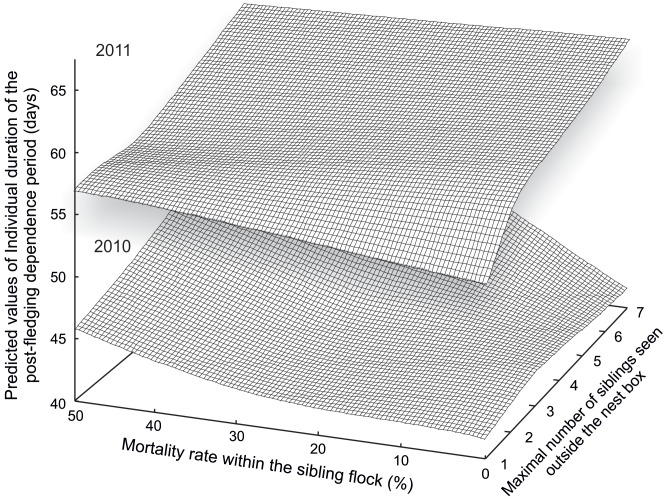
Predicted values of the individual duration of the post-fledging dependence period for both study seasons (2010 and 2011) plotted against the mortality rate (%, the number of dead fledglings as percentage of the total number of fledglings) within the sibling flock and the maximal number of siblings seen outside the nest box.

Individual duration of PFDP (Table 3) was dependent on maximal number of siblings seen outside the nest box nested within the year (Fig. 5), was associated with the mortality rate within the sibling flock in 2010, but not in 2011, and with interaction between the maximal number of siblings seen outside the nest box and the mortality rate within the sibling flock nested within the year (Fig. 6). The individual PFDP duration increased with increasing maximal number of siblings seen outside the nest box; mortality within the sibling flock also increased with maximal sibling number in both years. This interaction between maximal number of siblings seen outside the nest box and mortality rate within the sibling flock was more pronounced in 2010 than in 2011 (Fig. 6).

**Table 3:** The results of the GLMM for the Individual duration of PFDP.

Fixed effect	Num DF	Den DF	F value	P<
Maximal number of siblings seen outside the nest box nested within the year	2	21	19.5	0.0001
Mortality rate within the sibling flock nested within the year	2	21	2.95	0.074
Interaction between the maximal number of siblings seen outside the nest boxand the mortality rate within the sibling flock nested within the year	2	21	5.06	0.0016

## Discussion

To the best of our knowledge, this is the first study evaluating fledgling movement patterns of an owl species during two successive years with different prey availability (being high in 2010 while low in 2011). We found significant differences between the two seasons throughout the PFDP in the fledgling distances from the nest box, individual home range sizes throughout the PFDP and individual PFDP duration.

In the Tengmalm’s owl, egg laying is advanced, and clutch size and breeding success increases with the availability of main prey, i.e. different species of voles (*Arvicolidae*) and shrews (*Soricidae*) in Finland [62]–[64] and voles, shrews and mice (*Muridae*) in the Czech Republic [65]–[67]. We could not test the relationship between the variables measured in this study and prey availability directly, but because we have demonstrated differences between years in all variables analysed, we may speculate that these differences were indeed dependent on prey availability.

PFDP was almost doubled in the season with poor prey availability (53–61 days) than in the season with abundant prey (range 34–51 days). This is in accordance with the theory that predicts the length of PFDP should be maximal in years of poor food conditions [35]. The PFDP duration for individual chicks increased with increasing number of siblings seen outside the nest box and mortality rate within the sibling flock in both seasons. This may further suggest importance of food availability. More siblings in competition for the limited resources available to the providing male would delay growth and resulted both in an extension of the PFDP and a greater probability of mortality. Vergara et al. [50] hypothesised that under low food conditions, offspring may increase food solicitations to parents and thus increase food delivery. It seems that the parent’s ability to respond to chick’s begging behaviour depends on environmental circumstances, as suggested by our results of extended PFDP and data on begging behaviour in year with low prey availability. The length of PFDP is mainly an adult decision [50] and parents invest less (in terms of PFDP duration) when food is abundant, possibly because offspring may reach an optimal condition for independence earlier than when food is scarce which is supported by our results too. On the other hand the PFDP duration is partly dictated by the fledglings in Tengmalm’s owl since the young reached independence individually over an interval up to two weeks, generally in much the same order as that of date of hatching although with high inter-individual differences.

Such results contrasts with those reported for Tawny owl fledglings [34] where, within broods, the young stop begging synchronously independent of age differences, and mean independence age did not vary across years or between post-mast years and normal years. Thus parent-offspring conflict over the PFDP duration in Tawny and Tengmalm’s owls seems to be different. Ellsworth & Belthoff [68] observed that the independence of Western screech owl (*Megascops kennicottii*) fledglings follows systematically according to hatching date. We have found the same in Tengmalm’s owl fledglings (Kouba, unpublished data). Individual owlet characteristics (body mass at fledging and date of hatching, fledging and reaching independence) had no effect on individual PFDP duration in this study and our unpublished data thus support “early-dispersal hypotheses” - young individuals should stop begging while food is still offered and disperse as soon as ontogenetic development allows if the ability to gain earliest access to vital resources before other cohort members is the primary constraint on juvenile fitness after independence - suggested by Trivers [29], Nilsson [69] and Sunde [34] as opposite to “age-of-independence hypothesis” - if the duration of parental investment is important for future prospects of offspring, parent-offspring conflict likely arises as offspring matures and parents at some point cease providing food although offspring still wants feeding - observed in Tawny owl fledglings [34].

Contrary to what is the case for other owls, Tengmalm’s owl pairs commonly dissolve c. 20 days after the first owlet hatched [37], [38], [40]. Eldegard & Sonerud [41] have documented more recently that 70% of Tengmalm’s owl females had deserted the nest and brood by 34 days (median) after the first egg had hatched, and rather earlier in the case of food-supplemented nests. After leaving the nest, the female may choose to stay with nestlings and take part in food provisioning or abandon the brood absolutely and may start another brood with a new mate [41]–[44], [70]–[72]. In consequence, the great majority, if not all, of the prey delivered to juveniles throughout the late nestling and PFDP is provided by the male [42]–[44]. Thus, Tengmalm’s owl males have to take care of their brood in most cases independently from the female during the whole PFDP till reaching independence of the fledglings. This may cause an alternative pattern of the care over the young than that in species with permanent biparental care.

Our study documented that the distance between Tengmalm’s owl fledglings and their nest box increased gradually throughout the PFDP with the age of the fledglings. This pattern differed between the two years. Almost all fledglings moved more than 100 m from the nest box during the first week after last sibling fledged. Later on they were gradually moving away from the nest-site till the end of PFDP, reaching longer distances from the nest box and occupying larger home ranges in the season with lower prey availability (2011) compared to the season with higher prey availability (2010).

Our results are in accordance with our prediction that the offspring followed the provider to shorten the travel distance to the best hunting areas. We ourselves have no evidence as to presence or absence of the female in our study. But whichever parent is feeding the young, they must inevitably hunt at greater distances from the nest during low food season and thus, their fledglings follow them farther from the nest box too. This need seemed to be enhanced by increasing brood size in poor food years.

These results are in accordance with studies on other birds of prey. Gradual increase between fledglings and nest has been reported also by Penteriani et al. [28] and Delgado et al. [10] in Eagle owl, although they emphasise that the nest remained a focal point throughout the PFDP [as reported also by Belthoff & Ritchison [73] in Eastern Screech owl (*Megascops asio*), McClaren et al. [74] in Northern Goshawk (*Accipiter gentilis*) and Wood et al. [49] in Bald Eagle (*Haliaeetus leucocephalus*)]. In contrast Tengmalm’s owl fledglings completely abandoned the nest-site location soon after last individual fledged (1–2 weeks from fledging) and spent rest of the PFDP outside the nest-site.

Changes in distance of fledglings from the nest box with time were also dependent on the number of siblings present. In 2011 the distances from the nest box were found to increase with increasing number of siblings but in 2010, recorded distances decreased with brood size. It seems that if there are four or more fledged individuals, males may have a practical problem in leading the offspring in a certain direction from the nest box while keeping them close to each other.

Eldegard & Sonerud [42], [43] reported that members of any given brood in Tengmalm’s owl kept together until about four weeks after fledging and that the PFDP duration was 6–7 weeks. In our study area siblings stayed together throughout the PFDP, which lasted from 5–9 weeks. Recorded differences may be caused by different prey availability or due to the fact that the studies published by Eldegard & Sonerud [41]–[43] were based on tagging 1–2 fledglings from each nest.

We found noticeable differences in behaviour and movement patterns in Tengmalm’s owl fledglings throughout the PFDP compared to studies on fledglings of other, generally larger, owl species [10], [23], [28], [73]. However, post-fledging behaviour of another small owl species (the Northern Pygmy owl – *Glaucidium gnoma*) [75] is similar to that of the Tengmalm’s owl and suggests a consistent difference in strategy between larger species and these smaller owls.

We recorded fledglings during the PFDP up to two km from the nest box and Frye & Jageman [75] observed the young Northern Pygmy owls even further from the original nest location (3.3 km). These findings are remarkable because, for instance, offspring of Eagle owl were located only 1.5 km at their furthest from the nest through the entire PFDP [28] although they need five times more time to reach independence. It seems that this life-history trait may somehow be connected with overall small size of these study species; differences recorded may also be influenced by the degree of territoriality which is displayed, since this is usually stronger in bigger owl species [36], [39] while territorial behaviour was low or absent for Tengmalm’s owl and Northern Pygmy owl at least during the PFDP. These similarities in the behaviour of the fledglings of Tengmalm’s owl and the Northern Pygmy owl suggest different life-history strategies in parental care throughout the PFDP in these small owls by contrast to strategies reported for larger species.
